# Supplementary material for: Post kala azar dermal leishmaniasis and leprosy prevalence and distribution in the Muzaffarpur health and demographic surveillance site
Source: PLoS Negl Trop Dis. 2019 Oct 25;13(10):e0007798. doi: 10.1371/journal.pntd.0007798 (PMC6834282; doi:10.1371/journal.pntd.0007798)
Supplement: Attachment — Submitted filename: Comments_reviewers.docx [file pntd.0007798.s002.docx]

Dear editor,

We wish to thank the editor and the reviewers for constructive and positive commants received. We have made some changes in response, a point-by-point reply is provided below. We also uploaded a ‘track-changes’ version of the revised manuscript.

Please do not hesitate to contact us in case there are any further questions.

With kind regards,

Epco Hasker

Responses to the review comments and a description of the changes made in the manuscript:

Reviewer #1: Study objectives, methods, and ethics are clearly described. No comments.

Response: We thank the reviewer for this positive assessment.

Reviewer #2: Line 78 -79 is repeated in line 93-94

Response: We thank the reviewer for spotting this and changed the sentence in line 93-94 as follows: ‘Although as was stated earlier, PKDL is assumed to be less common on the Indian sub-continent than in East Africa, there are regional differences and in India in particular very few studies have been done’.

Reviewer #1: Study results are clearly presented, and matching the analysis plan. No comments.

Response: We thank the reviewer for this positive assessment.

Reviewer #2: Line 201: Only 4 PKDL suspects – this is quite a low number traditionally from ACD programmes that target PKDL, but may reflect the expertise of the physician and/or the lack of misdiagnosis with leprosy.

Response: Indeed, as mentioned in lines 20-21, the study took place in a Health and Demographic Surveillance Site (HDSS). The staff involved are very familiar with VL and PKDL. No changes made.

Reviewer #1: Authors fail to discuss the possible biases in the prevalence estimates, due to sub-optimal performance of the diagnostic confirmation tests used, or to the significant underreporting of adult males in the survey. This may have resulted in an underestimation of the prevalence estimates.

Response: We assume this comment is about PKDL, not about leprosy. As stated in line 159-160, ‘PKDL was confirmed by demonstration of parasites by microscopy in a skin smear or biopsy or by qPCR’. We agree with the reviewer that sensitivity may be an issue, however there were only four suspects so this cannot have had much influence on the prevalence estimate. We also agree with the reviewer that the composition of our study population may have been a source of bias. We added the sentence: ‘A limitation of our current survey is the fact that young adult men are under represented’ (line 258-259).

Reviewer #2: Out of interest were the 276 cases of previous VL found during the survey?

Response: The study area is a health and demographic surveillance site, details are described in reference 14, Malaviya et al. VL cases are reported on a continuous basis and annual surveys are conducted as well. No changes made.

Reviewer #1: I would like to congratulate the authors with this excellent study. This community survey elucidates the current prevalence of two important skin diseases, which are subject to elimination, and based on a spatial analysis suggests options for case finding strategies to support elimination.

Response: We thank the reviewer for these very encouraging comments.

Although I fully agree with the conclusion that a more in-depth cost-effectiveness study from the health system perspective is needed, the discussion on possible case finding strategies remain a bit thin. Authors suggest skin camps as an efficient and effective case finding strategy, however, only less than 2% of the people attending the skin camps were diagnosed with either leprosy or PKDL. Skin camps are a very resource intensive investment, which would have to be organised with a regular frequency in order to pick up the relatively low number of PKDL and leprosy cases. Should this be a priority in resource allocation for public health in a state, which is suffering from dire poverty and more important public health problems. Moreover, individuals with mild (macular) PKDL lesions may chose not to attend the skin camps, and may decline the treatment, which is long and with significant side effects.

Response: We agree with the reviewer that conducting skin camps for diagnosing just leprosy and PKDL may not be the most appropriate way of allocating resources. We advocate for addressing the basic needs of the population as stated in lines 288-289: ‘Most attendees, however, do not attend with complaints caused by PKDL or leprosy but because of other much more prevalent skin conditions. Many of those can be treated with relatively inexpensive means’ and in lines 292-294: ‘Responding to the basic health care needs of the population and seizing the opportunity to also search for PKDL and leprosy at the same time could be the most efficient way forward’. No changes made.

Authors suggest using the ASHAs network in case finding, but do not discuss what role these community health workers could play in active case finding strategies and to what extent they can make clinical (suspect) diagnoses, or how an additional task for skin diseases would compete with other priority tasks in the workload of the ASHAs.

Response: Indeed we have not described in detail the role ASHAs could play. We did make a reference to another paper by Malaviya et al. (ref. 21) in which we describe in more detail the potential role of ASHAs in VL and PKDL case finding and management. No changes made.

In view of the clear case clustering for leprosy, a strategy of door-to-door screening in the immediate neighborhood of identified cases could be cost-effective, considering the relatively high prevalence of the disease. However, for PKDL I would like to challenge authors to conclude that there may not be a cost-effective and sustainable case finding strategy, and that this has implications for sustainable VL elimination.

Response: We agree with the reviewer that even a few cases of PKDL could have serious implications for sustainable VL elimination. In line 272-273 we state that: ‘A few untreated PKDL patients in a non-immune population can be sufficient to trigger a new VL outbreak’. Indeed skin camps alone may not be the best strategy to detect PKDL. We therefore added to the conclusion that: ‘For PKDL this should be combined with active follow-up of previously treated VL cases’ (line 305-306).

Reviewer #2: Very clear and concisely written paper. Its true that the main limitation of this is validity in other settings that are not so well controlled, but at the same time the incidence and pathophysiological evolution of PKDL should not be much different to elsewhere. Well done!

Response: We thank the reviewer for this very positive assessment. We fully agree on the limitation on generalizability mentioned by the reviewer and mentioned also in the manuscript. No changes made.
